# The Effects of Early-Life Predator Stress on Anxiety- and Depression-Like Behaviors of Adult Rats

**DOI:** 10.1155/2014/163908

**Published:** 2014-04-15

**Authors:** Lu-jing Chen, Bing-qing Shen, Dan-dan Liu, Sheng-tian Li

**Affiliations:** ^1^Bio-X Institutes, Shanghai Jiao Tong University, Dongchuan Road 800, Shanghai 200240, China; ^2^Zhiyuan College, Shanghai Jiao Tong University, Dongchuan Road 800, Shanghai 200240, China; ^3^School of Life Science and Biotechnology, Shanghai Jiao Tong University, Dongchuan Road 800, Shanghai 200240, China

## Abstract

Childhood emotional trauma contributes significantly to certain psychopathologies, such as post-traumatic stress disorder. In experimental animals, however, whether or not early-life stress results in behavioral abnormalities in adult animals still remains controversial. Here, we investigated both short-term and long-term changes of anxiety- and depression-like behaviors of Wistar rats after being exposed to chronic feral cat stress in juvenile ages. The 2-week predator stress decreased spontaneous activities immediately following stress but did not increase depression- or anxiety-like behaviors 4 weeks after the stimulation in adulthood. Instead, juvenile predator stress had some protective effects, though not very obvious, in adulthood. We also exposed genetic depression model rats, Wistar Kyoto (WKY) rats, to the same predator stress. In WKY rats, the same early-life predator stress did not enhance anxiety- or depression-like behaviors in both the short-term and long-term. However, the stressed WKY rats showed slightly reduced depression-like behaviors in adulthood. These results indicate that in both normal Wistar rats and WKY rats, early-life predator stress led to protective, rather than negative, effects in adulthood.

## 1. Introduction

Many psychological diseases in adulthood are related to emotional traumas experienced during juvenility, especially depression and anxiety [1, 2]. For example, post-traumatic stress disorder (PTSD), also called post-traumatic stress reaction, is a severe anxiety disorder that develops after a person has experienced an acute, or chronic, stressor or traumatic event [3–5]. Examples of these types of stressors include natural disasters, such as floods, fires, and earthquakes. Also included are man-made traumatic events, such as physical assaults, motor-vehicle accidents, physical and sexual abuse, and witnessing violence, which produce intense negative feelings of fear or helplessness in the observer or participant [6]. Among the psychological traumas, childhood emotional trauma contributes significantly to some psychopathologies, such as PTSD. Epidemiological studies indicate that early-life stress, such as child abuse, is predominantly associated with a higher prevalence of a range of psychopathologies, particularly anxiety and depression [1, 7–12]. Therefore, it is important to study the effect of early-life psychological trauma on depressive and anxiety behaviors in adulthood.

Due to the ethical and practical limitations of applying psychological trauma on human beings, animal models are of great importance in studying the effects of psychological trauma. Over the past decades, a variety of valid animal models have been proposed for the study of stress and its effects [13–21]. Many different stressors were used in those studies, such as maternal separation, predator stress, and drug stimulation [18, 22, 23]. Predator stress is one of the most widely used stressors. Several reports have shown that rats and mice displayed anxiety- and depression-like behaviors in the short-term after exposure to predator scent or a predator per se [7, 13, 24–27]. Very few studies, however, have focused on long-term behavioral changes after predator stress. By using “cut-off behavioral criteria” (which allow for discrimination between clusters of behaviors analogous to anxious and depressive states), Tsoory et al. showed that cat urine exposure in juveniles induced anxiety- and depression-like behaviors in adulthood [7]. Miura et al. showed, however, that predator stress could reduce the level of anxiety in both the short-term (one week) and long-term (four weeks) [12]. In Miura's research, rats were chosen as the predators of mice; thus, we cannot determine whether these outcomes resulted from the differences in the choice of predator (rat or cat). Thus, the effects of predator stress are still not clearly identified. In the current experiment, we focused on predator stress during juvenility. Since some key brain areas involved in emotion, such as the prefrontal cortex, hippocampus, and amygdale, are still undergoing significant maturation processes during this period [28], juvenile stress may affect brain development and may have significant effects even in adulthood. We modeled childhood psychological trauma by exposing juvenile Wistar rats to a feral cat to study whether or not predator stress contributes to anxiety- or depression-like behaviors in adulthood. In addition, it is known that WKY rats exhibit endogenous anxiety- and depression-like behaviors and have been proposed as an animal model for depression-like behavior [29–32]. However, the effect of predator stress on WKY rats has not been studied yet. Since Miura's research showed some protective effects of predator stress [12], we also exposed juvenile WKY rats to the same predator stress as Wistar rats to study whether early-life predator stress can affect the depression-like behavior of WKY rats.

## 2. Materials and Methods

### 2.1. Animals

All experiments were conducted according to the Health Guide for the Use and Care of Laboratory Animals of Shanghai Jiao Tong University. Male Wistar rats (3 weeks old) and male Wistar Kyoto (WKY) rats (3 weeks old) were purchased from Vital River Laboratory Animal Technology Co., Ltd., in Beijing. All rats were maintained on a 12-hr light-dark cycle with lights on at 12 a.m. and lights off at 12 p.m., at room temperature 22 ± 2°C, and at room humidity around 40%. Each rat was housed in a separate cage. WKY rats are required to be housed separately to induce the depression-like behavior; in order to reduce the effect caused by housing conditions, we housed Wistar rats separately as well. After one-week adaptation, rats (4 weeks old) were randomly divided into four groups: Wistar control, Wistar predator stress, WKY control, and WKY predator stress groups; each group consisted of 5 rats. The mean body weights of each group were 113.9 ± 2.3 g, 118.9 ± 4.6 g, 98.2 ± 2.5 g, and 96.8 ± 3.6 g, respectively. All the animals were treated alike except for the predator exposure between the control and stress groups. During the experiment, we recorded the body weight of each rat, both before and after predator stress. The percentage of weight change was calculated as (weight after predator stress—weight before predator stress)/weight before predator stress ∗ 100%.

### 2.2. Predator Stress

**Figure 1:**
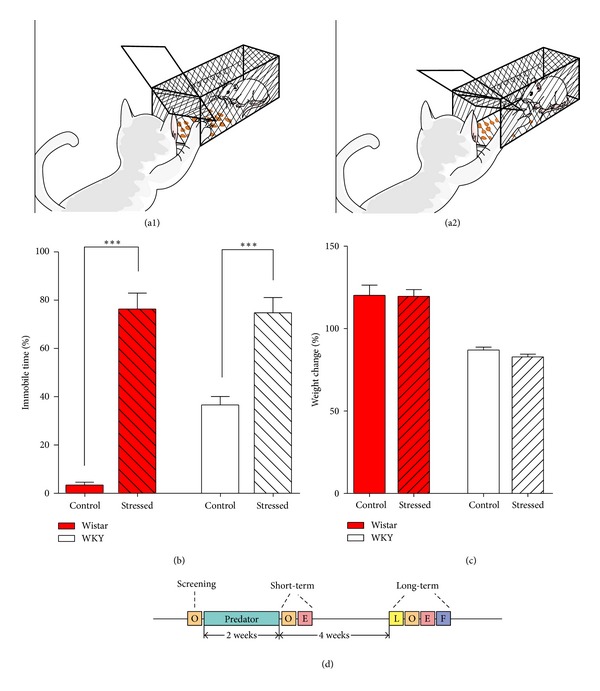
(a1) and (a2) procedure of predator stress. The feral cat could open the door to get the food inside, which intensely stressed the rat inside. The cage and the door were not large enough to allow the cat to fully enter, avoiding actual contact between the cat and the rat. Stimulations lasted for 10 min every day for 2 weeks. (b) Changes in the percent of freezing time of rats when exposed to predator stress. The percent of freezing time increased significantly while being exposed to a predator in both strains. Note that the control group of Wistar and WKY rats also showed differences in the percentage of freezing time. (c) Weight changes of rats before and after 2-week predator stress. Statistical differences only existed between the strains, not within the strains. Each bar indicates a group defined according to strains (Wistar rats and WKY rats) and predator exposure (red bar: Wistar rats; white bar: WKY rats; unshadowed bar: control group; shadowed bar: predator exposure). Values are shown as mean ± SEM. The results of* t*-test are shown. ***P < 0.001 for the effects of predator stress. (d) Procedure of experiment. O: open field test; E: elevated plus maze test; L: light/dark transition test; F: forced swim test; Predator: predator stress.

A healthy adult feral cat caught from our campus was used as the predator for rats. When being exposed to a predator, each rat was placed in a black cage, constructed by experimenters, with doors that can be opened toward the inside. Food was placed inside the cage, and the door was linked to a spring that kept the cage closed but could be opened when the cat pushed it to get the food. The door and the cage were not large enough to allow the cat entry; this avoided actual contact between the cat and the rats. After training, the cat could reach its claws into the cage to obtain the food. Before each stress, the rats were taken out for 30 min, and their weights were obtained. During the predator stress, an individual rat was put into each cage, and the cat was allowed to acquire the food inside the cages for 10 min (Figures 1(a1) and 1(a2)). Predator stress was given when the rats were 4 weeks old. The predator stress was applied 10 min per day during the dark cycle at room temperature 24 ± 1°C for 14 consecutive days. The exact time of each predator stress varied from day to day. All of the animals in the stress groups were exposed to the predator at the same time every day. The control animals were placed in another room under the same conditions except for the predator exposure.

### 2.3. Behavioral Tests

All behavioral tests were performed during the dark cycle at room temperature 24 ± 1°C. Each test started from around 6 p.m. and ended around 9 p.m. We performed a pre-open field test before predator stress to exclude some abnormal animals; an open field test and elevated plus maze test shortly following predator stress; and a light/dark transition test, open field test, elevated plus maze test, and forced swim test sequentially 4 weeks after the predator stress. The order of these tests is shown in Figure 1(d). Considering that the open field test would take a very long time if all of the animals were tested on the same day, we conducted an open field test on two consecutive days, with half of the control animals and half of the predator-stressed animals on each day. For all other experiments, we tested all of the animals on the same day. All the behavioral tests were recorded using a video camera and analyzed from the videotapes by using a motion tracking system (R.D, provided by MobileDatum Co., Ltd., Shanghai, China).

### 2.4. Open Field Test

The open field apparatus was a soundproof box with an uncovered square box (40 × 40 × 45 cm) inside. The inside area of the apparatus was black, and testing was conducted under dim light. The rat was placed in the area with its head toward the inside of the box and allowed to behave freely for 10 min. During the test, rearing times and face-washing times were counted by the experimenters, while other data, including total distance and time in the center square, were analyzed by the motion tracking system. After each trial, the rat was returned to its home cage, and the square box was cleaned with 75% ethanol and dried with an air drier.

### 2.5. Elevated Plus Maze Test

The elevated plus maze consisted of two open arms (50 × 10 cm), two closed arms (50 × 10 cm), a central platform (10 × 10 cm), and 10 cm high walls that enclosed the closed arms. The four arms were 70 cm high from the floor, and the inside was completely black. A ceiling-mounted video camera recorded each trial. Each rat was placed into the center of the maze with its head toward the open arms and was allowed to freely explore for 5 min. When more than half of a rat's body entered an arm, it was considered to be inside the arm. After each trial, the rat was returned to its home cage, and the floor and the walls of the maze were cleaned with 75% ethanol and dried with an air drier. Entry times to each arm and time spent on each arm were analyzed by the motion tracking system, as mentioned above. Entries into open arms (%) and time on open arms (%) were concomitantly calculated as the behavioral indexes of anxiety.

### 2.6. Light/Dark Transition Test

The apparatus consisted of a box (21 × 42 × 25 cm) and a video camera. A wall inside of the box separated the box into two rooms: the light box and the dark box. The two boxes were of the same size. The light box was illuminated by a white LED, while the dark box was illuminated by an infrared lamp. The roof of the apparatus could be opened in order to place the rat inside. At the beginning of the test, an individual rat was placed in the light box and was allowed to explore the boxes freely for 10 min. Behaviors were recorded by two infrared video cameras placed in the ceilings of the rooms. The latency of transmitting from the dark box to the light box, the time spent in each box, the number of transitions, and the total distance traveled were calculated and analyzed by the motion tracking system, as mentioned above. After each trial, the rat was returned to its home cage, and the two boxes were cleaned with 75% ethanol and dried with an air drier.

### 2.7. Forced Swim Test

The equipment of the forced swim test consisted of a cylindrical water tank (diameter = 30 cm and height = 45 cm) and a video camera placed horizontally on the side. During each trial, the tank was filled with 25 ± 1°C water up to 30 cm height. On the first day, an individual rat was placed in the water tank for 10 min to freely explore and dive. 24 h later, the rats were again placed in the water for 5 min, and the duration of immobility behavior (floating in the water without struggling) was measured by the motion tracking system mentioned above, as one of the indexes of depression-like behaviors. The rat was then removed from the water, dried with a towel, and returned to its home cage after each trial.

### 2.8. Statistical Analyses

Behavioral data were shown as mean ± SEM. Statistical comparisons between the control and stressed group in each strain were determined by using *t*-test with the alpha level set at 0.05. The differences between strains were determined by using 2 × 2 ANOVA test with the alpha level set at 0.05. All of the data were plotted using Prism software.

## 3. Results

### 3.1. Predator Stress

The open field test is well known to reflect anxiety- and depression-like behaviors of rats and mice [33]. Therefore, we used the open field test to screen all rats, in order to reduce individual variation prior to predator stress. The differences in total distance, rearing times, and face-washing times of all rats were analyzed. Results showed that only one Wistar rat's behaviors were abnormal in all of the indexes, although statistical differences did not exist between groups (see Supplemental Figure 1 in Supplementary Material available online at http://dx.doi.org/10.1155/2014/163908). Thus, we excluded this Wistar rat from further experiments.

The responses of rats to predator stress have often been evaluated by changes of self-directed behaviors, such as face-washing and sniffing [34]. Since the rats receiving predator stress in our experiment showed immobile response (an absence of obvious movements of the head and body) most of the time, we only calculated the percentage of immobile time during predator stress. The predator stress in our experimental condition did produce severe emotional stress in the rats, as indicated by the Wistar rats showing over 25 times the immobile behavior during predator stress compared to the same group before stress (Figure 1(b), before: 3.4 ± 1.2%, *N* = 4; stressed: 80.5 ± 6.5%, *N* = 4; *P* < 0.001). WKY rats, however, demonstrated two times the immobile behavior compared to the same group before stress (Figure 1(b), before: 36.6 ± 3.5%, *N* = 5; stressed: 74.8 ± 6.3%, *N* = 5; *P* < 0.001). We also calculated weight changes before and after the 2-week predator stress; no significant differences in weight were found between the control group and stressed group before and after stress in both Wistar (control: 120.3 ± 6.1%, *N* = 5; stressed: 119.7 ± 4.0%, *N* = 4; *P* > 0.05) and WKY rats (control: 87.0 ± 1.8%, *N* = 5; stressed: 82.9 ± 1.7%, *N* = 5; *P* > 0.05) (Figure 1(c)).

### 3.2. Short-Term Effects of Predator Stress

**Figure 2:**
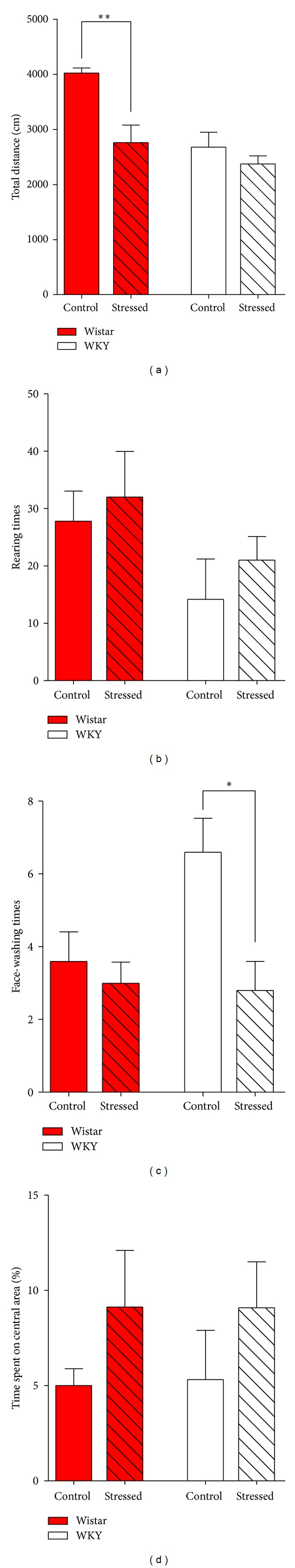
Total distance (a), rearing times (b), face-washing times (c), and percent of time spent on central area (d) in open field test in the short-term (two days) after 2-week predator stress. The stimulation decreased total distance traveled by Wistar rats (a) and face-washing times of WKY rats (c), respectively. Note that, compared to control Wistar rats, control WKY rats showed significantly decreased total distance (a) and increased washing times (c). Each bar indicates a group defined according to strains (Wistar rats and WKY rats) and predator exposure (red bar: Wistar rats; white bar: WKY rats; unshadowed bar: control group; shadowed bar: predator exposure). Values are shown as mean ± SEM. The results of* t*-test are shown. *P < 0.05; **P < 0.01 for the effects of predator stress.

The short-term effects of predator stress on anxiety- and depression-like behaviors were evaluated by the open field test and the elevated plus maze test on the second day after the 2-week predator stress. Considering the high level of stress induced by the forced swim test per se, we did not perform it at this time in order to avoid a confounding effect on the adult rats' behaviors. In the open field test, predator-stressed Wistar rats showed significantly shorter total distance traveled compared to control Wistar rats (stressed Wistar, 2795.0 ± 319.7 cm, *N* = 4; control Wistar, 4024.0 ± 91.7 cm, *N* = 5; *P* < 0.01) (Figure 2(a)), while the rearing times, face-washing times, and the time spent in the central area remained unchanged (Figures 2(b)–2(d) and Supplemental Table 1). On the other hand, stressed WKY rats showed significantly less face-washing times compared to control WKY rats (Figure 2(c), stressed WKY rats, 3.6 ± 0.8, *N* = 5; control WKY rats, 6.6 ± 0.9, *N* = 5; *P* = 0.0146), while no significant differences were shown on other indexes (Figures 2(a)-2(b), 2(d), and Supplemental Table 1). In the elevated plus maze test, we calculated the percentage of time spent on the open arms and the percentage of entry times into the open arms; neither of the two indexes differed significantly between the control and the stimulated Wistar or WKY rats (Supplemental Table 2). In summary, 2-week predator stress decreased the total distance traveled of Wistar rats and face-washing time of WKY rats, respectively, in the open field test.

### 3.3. Long-Term Effects of Predator Stress

**Figure 3:**
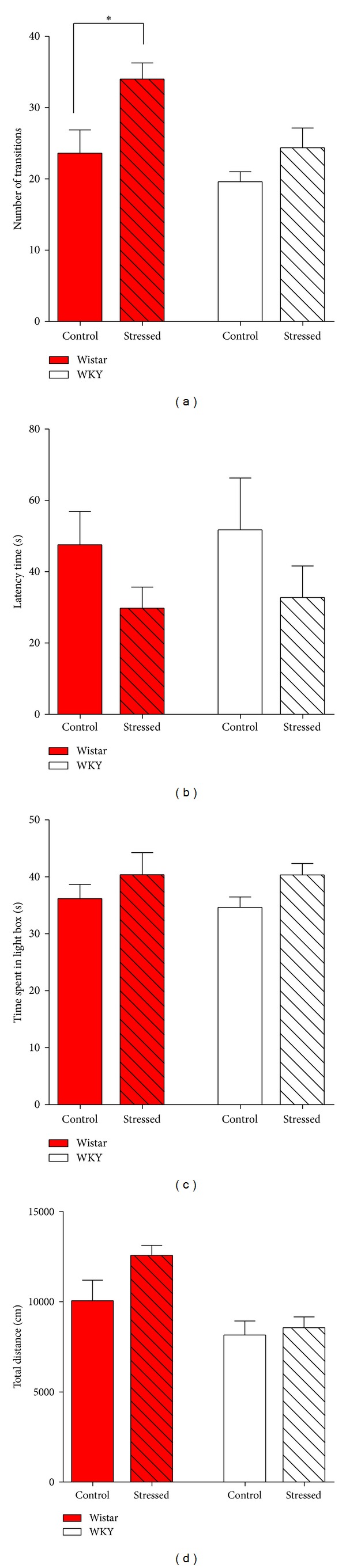
Behaviors of rats in light/dark transition test in the long-term (30 days) after 2-week predator stress. Wistar rats that received early-life predator stress showed significantly more number of transitions compared with control Wistar rats (a), while there were no obvious differences in latency (c), total time spent in the light box (d), and total distance between stimulated and control groups (b)–(d). Each bar indicates a group defined according to strains (Wistar rats and WKY rats) and predator exposure (red bar: Wistar rats; white bar: WKY rats; unshadowed bar: control group; shadowed bar: predator exposure). Values are shown as mean ± SEM. The results of* t*-test are shown. *P < 0.05 for the effects of predator stress.

**Figure 4:**
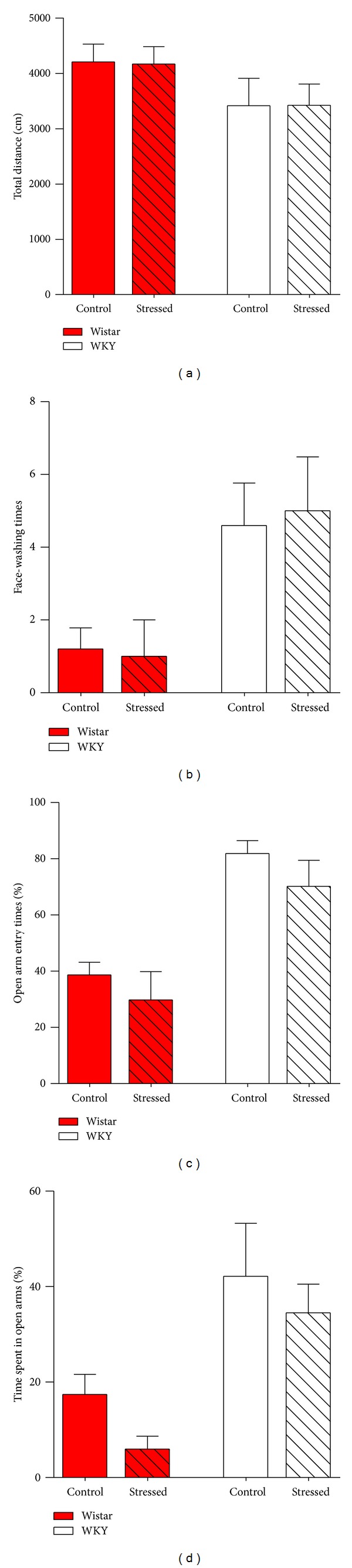
Behaviors of rats in open field test and elevated plus maze test in the long-term (30 days) after 2-week predator stress. (a) Total distance in open field test. (b) Face-washing times in open field test. (c) Percent of open arm entries in elevated plus maze test. (d) Percent of time spent on open arms in elevated plus maze test. No significant differences were found in the above tests between stimulated and control Wistar or WKY rats. Note that control WKY rats showed more face-washing times in open field test (b) and more open arms entry times in elevated plus maze test (c) compared with control Wistar rats. Each bar indicates a group defined according to strains (Wistar rats and WKY rats) and predator exposure (red bar: Wistar rats; white bar: WKY rats; unshadowed bar: control group; shadowed bar: predator exposure). Values are shown as mean ± SEM. The results of* t*-test are shown.

**Figure 5:**
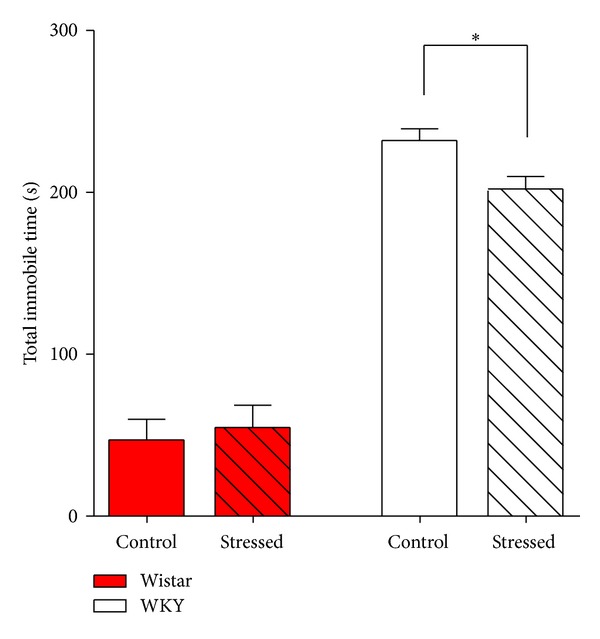
Immobile time of rats in forced swim test in the long-term (30 days) after 2-week predator stress. The immobile time (floating without struggling) of stimulated WKY rats was significantly lower than that of the control WKY rats, while Wistar rats showed no changes between predator-stimulated and control groups. Note that control WKY rats showed significantly longer immobile time than control Wistar rats. Each bar indicates a group defined according to strains (Wistar rats and WKY rats) and predator exposure (red bar: Wistar rats; white bar: WKY rats; unshadowed bar: control group; shadowed bar: predator exposure). Values are shown as mean ± SEM. The results of* t*-test are shown. *P < 0.05 for the effects of predator stress.

Early-life stress, such as child abuse, is predominantly associated with a higher prevalence of a range of psychopathologies, particularly anxiety and depression [1, 7–12]. Thus, we next examined the long-term effects of early-life predator stress on anxiety- and depression-like behaviors of both Wistar and WKY rats 4 weeks after the predator stress. The light/dark transition test, open field test, elevated plus maze test, and forced swim test were performed sequentially. The light/dark transition test is used to test anxiety-like behaviors of rats and mice by analyzing the latency to enter the light box, the percentage of time spent in the light box, the number of transitions, and the total distance traveled [35, 36]. As shown in Figure 3(a), Wistar rats that received early-life predator stress (34.0 ± 2.3, *N* = 4) showed significantly more transit times compared with control Wistar rats (23.6 ± 3.3, *N* = 5; *P* = 0.0432), whereas there were no obvious differences in the mean latency of entering into the light box, time spent in the light box, and total distance between stressed and control Wistar or WKY rats (Figures 3(b)–3(d) and Supplemental Table 3). Similarly, neither the open field test nor the elevated plus maze test showed statistical differences between control and stressed Wistar or WKY rats (Figures 4(a)–4(d) and Supplemental Tables 4-5). In the forced swim test, which is a canonical behavioral test used to assess depression-like behaviors of rats and mice [37, 38], the immobile time (floating without struggling) of stressed WKY rats (202.0 ± 7.8 s, *N* = 5) was significantly lower than that of control WKY rats (232.0 ± 7.5 s, *N* = 4; *P* = 0.0295), whereas Wistar rats showed no changes between the predator-stressed and control groups (Figure 5 and Supplemental Table 6). In summary, early-life predator stress increased the number of transitions of Wistar rats in the light/dark transition test and decreased immobile times of WKY rats in the forced swim test.

## 4. Discussion

### 4.1. Short-Term Effects of Predator Stress

In the current study, in order to investigate the effect of psychological trauma on anxiety- and depression-like behaviors, we applied 2-week predator stress (exposure to a feral cat) to rats (Wistar or WKY rats, 4 weeks old) and examined its effects immediately after the 2-week predator stress. Our results showed that predator stress decreased the total distance traveled by Wistar rats (Figure 2(a)) and reduced the face-washing times of WKY rats in an open field test (Figure 2(c)), respectively. This suggests that 2-week predator stress decreased spontaneous activities in Wistar rats immediately after stress but did not increase anxiety-like behaviors. Additionally, it has been reported that face-washing, as well as time spent in the central area in an open field test, represents anxiety-like behaviors [33, 39]. In addition to significantly reduced face-washing times, the predator-stimulated WKY rats also showed a tendency to increase time spent in the central area. However, no significant differences were shown in elevated plus maze test, the canonical test for anxiety-like behaviors, between control and stressed groups. This suggests that predator stress did not increase anxiety-like behaviors but had some slightly protective effects. These results differ from many previous reports [7, 13, 24–27] that have indicated that predator stress increased anxiety-like behaviors. One possible explanation for this discrepancy is the intensity of the predator stress. In our experiment, we trained the feral cat to be able to open the door of the cage and extend its paws deep into the cage, thereby intensely stressing the rat inside the cage; this was reflected by the Wistar rats showing an increase of over 25 times in immobile time during predator stress (from 3% to 80%, Figure 1(b)). Conversely, in previous research, mice showed more active behaviors, such as grooming and sniffing, rather than an immobile response, when exposed to a predator (rat) [34]. Thus, the discrepancy in the effects of predator stress on anxiety- and depression-like behaviors may be due to different intensities of predator stress.

### 4.2. Long-Term Effects of Predator Stress

The long-term effects of early-life predator stress on anxiety- and depression-like behaviors of both Wistar and WKY rats were examined 4 weeks after the end of the predator stress. Our results showed that early-life predator stress increased the number of transitions of adult Wistar rats in the light/dark transition test (Figure 3(a)) and decreased immobile time of adult WKY rats in the forced swim test (Figure 5). There were no obvious differences, however, in other indexes representing anxiety-like behaviors between control and predator-stimulated Wistar or WKY rats (Figures 3(b)–3(d), Figures 4(a)–4(d), and also Supplemental Tables 3–5). Taken together, these results indicate that early-life predator stress did not increase anxiety-like behaviors in adult Wistar rats and has some, although not large, protective effects. Predator stress also caused a slight decrease in the level of depression-like behaviors in adult WKY rats. Some reports have shown that predator stress could increase long-lasting (about three weeks) anxiety-like behaviors [13, 20, 21]. The most plausible explanation for the opposite long-term effect of predator stress is the intensity of predator stress, as discussed above. Interestingly, in some clinical studies, some people who suffered from psychological trauma showed posttraumatic growth [40]. Our research can be an example of posttraumatic growth on animals.

On the other hand, due to unforeseen loud noise caused by construction activity in our upstairs laboratory, all of the animals in our experiment unavoidably experienced stressful conditions from the 20th to 29th day after the end of the predator stress (it continued for 9 days before the final behavioral test battery) and may have affected the results of the behavioral tests. Nevertheless, our results still suggest that juvenile predator stress at least did not enhance depression- or anxiety-like behaviors and might provide some protective effects.

### 4.3. Different Effects of Predator Stress between Wistar and WKY Rats

In terms of the short-term effects, predator stress increased depression-like behaviors (decreased total distance traveled in the open field test) in Wistar rats, but not in WKY rats (Figure 2(a)). In contrast, regarding the long-term effects, predator stress relieved depression-like behaviors in the forced swim test only in WKY rats, but not in Wistar rats. Considering that WKY strain is a model for depression-like behaviors, we used two-way ANOVA to test the differences between the two strains. Results showed that statistical differences exist between Wistar and WKY rats in many indexes (Supplemental Tables 7–9). These differences may contribute to the different results of behavioral tests between the two strains. Thus, the mechanisms underlying the different effects of predator stress on anxiety- and depression-like behaviors between Wistar and WKY rats require further study into the basis of the induction mechanism of depression in WKY rats.

## 5. Conclusions

In the current study, we show that, in Wistar rats, early-life predator stress decreased spontaneous activities in the short-term after the stimulation but did not increase anxiety- or depression-like behaviors in adulthood. In the genetic depression model rats, the WKY rats, the same early-life predator stress did not increase anxiety- or depression-like behaviors in adulthood as well. However, the predator stress slightly decreased anxiety-like behavior in Wistar rats and decreased depression-like behavior in WKY rats in adulthood. These results suggest that early-life predator stress, at least for rats, did not induce PTSD but may result in posttraumatic growth in adulthood. Further investigations are required to elucidate the neurobiological underpinnings of the effects of early-life predator stress on anxiety and depression-like behaviors.

## Supplementary Material

Supplementary materials describe the results of our behavioral tests, including light/dark transition test, open field test, elevated plus maze test and forced swim test. We listed the mean value of each index and the P value of statistical analysis. Additionally, we also included the result of our screening experiment in Supplemental Figure 1, which is a pre-open field test used for screening some abnormal animals before final experiment.
