# Supplementary material for: The Effects of Early-Life Predator Stress on Anxiety- and Depression-Like Behaviors of Adult Rats
Source: Neural Plast. 2014 Apr 15;2014:163908. doi: 10.1155/2014/163908 (PMC4009288; doi:10.1155/2014/163908)
Supplement: Supplementary file 1 — Supplementary materials describe the results of our behavioral tests, including light/dark transition test, open field test, elevated plus maze test and forced swim test. We listed the mean value of each index and the P value of statistical analysis. Additionally, we also included the result of our screening experiment in Supplemental Figure 1, which is a pre-open field test used for screening some abnormal animals before final experiment. [file 163908.f1.docx]

|  | **Wistar(s), N = 4** | **Wistar(c), N = 5** | **p value** |
| --- | --- | --- | --- |
| total distance (cm) | 2795.0 ± 319.7 | 4024.0 ± 91.7 | 0.0039^*^ |
| rearing times | 32.0 ± 8.0 | 27.8 ± 5.3 | 0.6623 |
| face washing times | 3.0 ± 0.6 | 3.6 ± 0.8 | 0.5861 |
| distance on central area (%) | 12.4 ± 3.8 | 5.6 ± 1.5 | 0.1154 |
| time on central area (%) | 9.1 ± 3.0 | 5.0 ± 0.9 | 0.1848 |
|  | **WKY(s), N = 5** | **WKY(c), N = 5** | **p value** |
| total distance (cm) | 2373.0 ± 147.5 | 2678.0 ± 269.0 | 0.3495 |
| rearing times | 21.0 ± 4.1 | 14.2 ± 7.0 | 0.4277 |
| face washing times | 2.8 ± 0.8 | 6.6 ± 0.9 | 0.0146^*^ |
| distance on central area (%) | 11.3 ± 2.7 | 5.2 ± 2.0 | 0.1099 |
| time on central area (%) | 9.1 ± 2.4 | 5.3 ± 2.6 | 0.3175 |

Supplemental Table 1

Data of open field test in the short term (2 days) after 2-week predator stress. Effects of predator stress were analyzed by t-test. Results of t-test are shown. *: p < 0.05.

Supplemental Table 2

|  | **Wistar(s), N = 4** | **Wistar(c), N = 5** | **p value** |
| --- | --- | --- | --- |
| time on open arms (%) | 15.5 ± 4.0 | 28.8 ± 17.7 | 0.5353 |
| entries into open arms (%) | 31.2 ± 12.3 | 40.7 ± 16.4 | 0.6718 |
|  | **WKY(s), N = 5** | **WKY(c), N = 5** | **p value** |
| time on open arms (%) | 31.6 ± 4.5 | 42.3 ± 14.9 | 0.7709 |
| entries into open arms (%) | 55.0 ± 3.9 | 51.0 ± 10.1 | 0.722 |

Data of elevated plus maze test in the short term (2 days) after 2-week predator stress.

Supplemental Table 3

|  | **Wistar(s), N = 4** | **Wistar(c), N = 5** | **p value** |
| --- | --- | --- | --- |
| transit times | 34.0 ± 2.3 | 23.6 ± 3.3 | 0.0432^*^ |
| latency time (s) | 29.8 ± 5.9 | 47.6 ± 9.3 | 0.1724 |
| time in light box (%) | 40.4 ± 3.9 | 36.2 ± 2.5 | 0.3749 |
| total distance (cm) | 12566.0 ± 548.1 | 10050.0 ± 1149.0 | 0.1131 |
|  | **WKY(s), N = 5** | **WKY(c), N = 5** | **p value** |
| transit times | 24.4 ± 2.8 | 19.6 ± 1.4 | 0.1584 |
| latency time (s) | 32.8 ± 8.8 | 51.8 ± 14.5 | 0.2949 |
| time in light box (%) | 40.4 ± 2.0 | 34.7 ± 1.8 | 0.0684 |
| total distance (cm) | 8562.0 ± 596.3 | 8161.0 ± 772.2 | 0.6919 |

Data of light/dark transition test in the long term (30 days) after 2-week predator stress. Effects of predator stress were analyzed by t-test. Results of t-test are shown. *, p < 0.05.

Supplemental Table 4

|  | **Wistar(s), N = 4** | **Wistar(c), N = 5** | **p value** |
| --- | --- | --- | --- |
| total distance (cm) | 4167.0 ± 315.5 | 4005.0 ± 322.7 | 0.9367 |
| rearing times | 30.0 ± 10.2 | 29.8 ± 6.5 | 0.9867 |
| face washing times | 1.0 ± 1.0 | 1.2 ± 0.6 | 0.8608 |
| distance on central area (%) | 16.2 ± 2.7 | 10.1 ± 2.8 | 0.1718 |
| time on central area (%) | 15.0 ± 2.9 | 9.5 ± 2.7 | 0.2136 |
|  | **WKY(s), N = 5** | **WKY(c), N = 5** | **p value** |
| total distance (cm) | 3423.0 ± 381.6 | 3415.0 ± 497.2 | 0.9899 |
| rearing times | 31.8 ± 4.8 | 28.0 ± 9.8 | 0.7369 |
| face washing times | 5.0 ± 1.5 | 4.6 ± 1.2 | 0.8374 |
| distance on central area (%) | 6.7 ± 2.2 | 7.0 ± 2.5 | 0.9281 |
| time on central area (%) | 7.3 ± 2.4 | 7.5 ± 2.6 | 0.9668 |

Data of open field test in the long term (30 days) after 2-week predator stress. Effects of predator stress were analyzed by t-test.

Supplemental Table 5

|  | **Wistar(s), N = 4** | **Wistar(c), N = 5** | **p value** |
| --- | --- | --- | --- |
| time on open arms (%) | 6.0 ± 2.7 | 17.4 ± 4.2 | 0.0673 |
| entries into open arms (%) | 29.8 ± 10.1 | 38.7 ± 4.5 | 0.4109 |
|  | **WKY(s), N = 5** | **WKY(c), N = 5** | **p value** |
| time on open arms (%) | 34.5 ± 6.0 | 42.2 ± 11.1 | 0.5609 |
| entries into open arms (%) | 70.25 ± 9.2 | 81.9 ± 4.6 | 0.2928 |

Data of elevated plus maze test in the long term (30 days) after 2-week predator stress. Effects of predator stress were analyzed by t-test.

|  | **Wistar(s), N = 4** | **Wistar(c), N = 4** | **p value** |
| --- | --- | --- | --- |
| total immobile time (s) | 54.9 ± 13.6 | 47.1 ± 12.8 | 0.6914 |
| total mobile time (s) | 245.1 ± 13.6 | 252.9 ± 12.8 | 0.6914 |

Supplemental Table 6

|  | **WKY(s), N = 5** | **WKY(c), N = 4** | **p value** |
| --- | --- | --- | --- |
| total immobile time (s) | 202.0 ± 7.8 | 232.0 ± 7.5 | 0.0295^*^ |
| total mobile time (s) | 94.8 ± 10.7 | 67.99 ± 7.5 | 0.0931 |

Data of forced swim test in the long term (30 days) after 2-week predator stress. Effects of predator stress were analyzed by t-test. Results of t-test are shown. *, p < 0.05.

Supplemental Table 7

|  | **P value for strain** | **P value for strain*treatment** |
| --- | --- | --- |
| Immobile time during stress | 0.004^**^ | 0.000^***^ |

Two-way ANOVA results for total immobile time during predator stress. **, p < 0.01; ***, p < 0.001.

Supplemental Table 8

|  | **P value for strain** | **P value for strain*treatment** |
| --- | --- | --- |
| OFT-total distance | 0.001^**^ | 0.002^**^ |
| OFT-face washing times | 0.107 | 0.017^*^ |
| OFT-rearing times | 0.036^*^ | 0.762 |
| OFT-time on central area | 0.952 | 0.106 |
| EPM-open arm entry | 0.163 | 0.816 |
| EPM-time in open arm | 0.179 | 0.489 |

Two-way ANOVA results for short-term behavioral tests. OFT stands for open field test. EPM stands for elevated plus maze test. *, p < 0.05; **, p < 0.01.

Supplemental Table 9

|  | **P value for strain** | **P value for strain*treatment** |
| --- | --- | --- |
| L/D-number of transitions | 0.018^*^ | 0.010^*^ |
| L/D-time in light box | 0.759 | 0.069 |
| L/D-total distance | 0.003^**^ | 0.101 |
| L/D-latency time | 0.737 | 0.102 |
| OFT-total distance | 0.072 | 0.971 |
| OFT-face washing times | 0.005^**^ | 0.931 |
| OFT-rearing times | 1.000 | 0.805 |
| OFT-time on central area | 0.088 | 0.333 |
| EPM-open arm entry | 0.000^***^ | 0.179 |
| EPM-time in open arm | 0.002^**^ | 0.202 |
| FST-immobile time | 0.000^***^ | 0.311 |

Two-way ANOVA results for long-term behavioral tests. L/D stands for light/dark transition test. OFT stands for open field test. EPM stands for elevated plus maze test. FST stands for forced swim test. *, p < 0.05; **, p < 0.01, ***, p < 0.001.


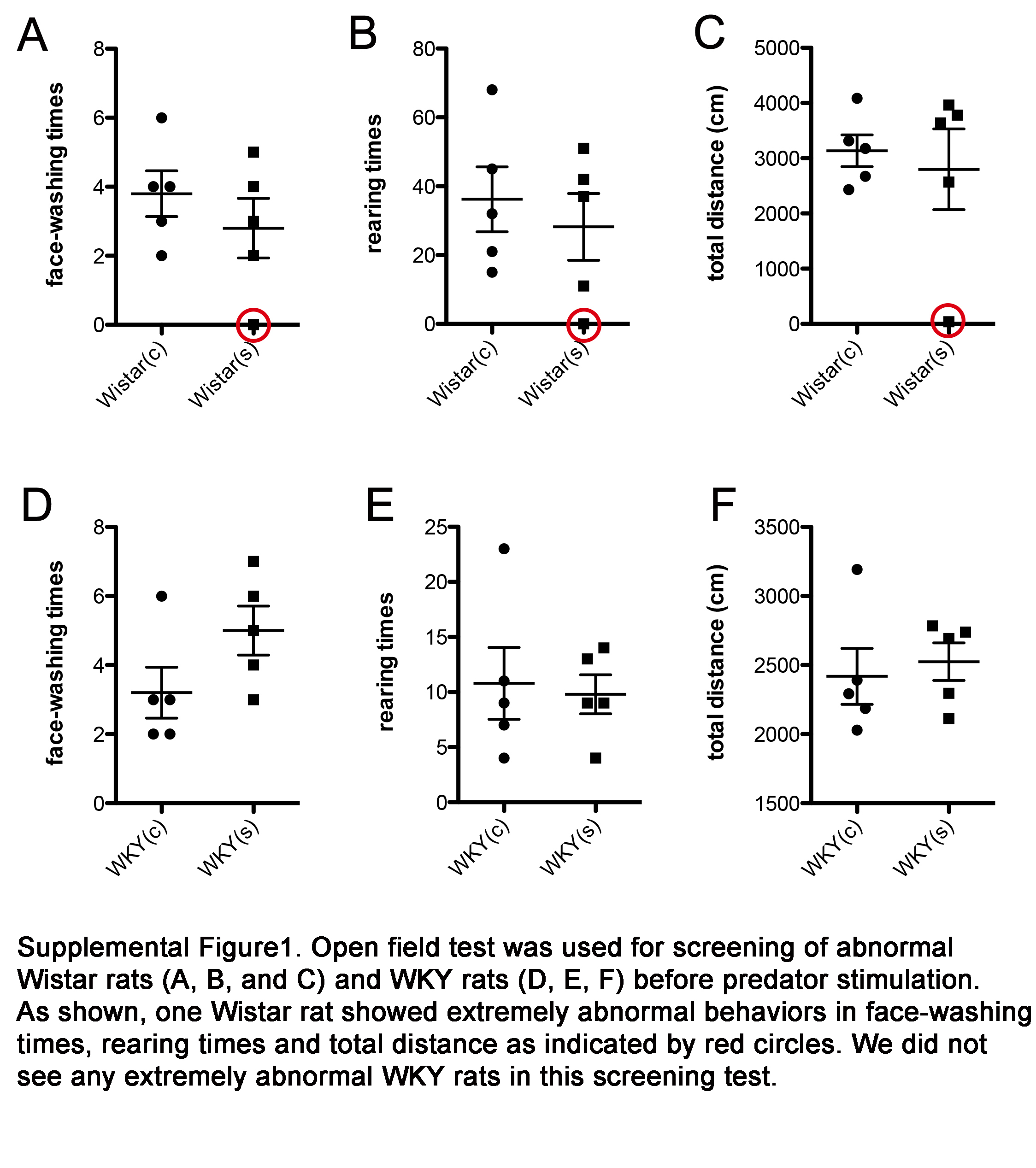


Supplemental Figure1. Open field test was used for screening of abnormal Wistar rats (A, B, and C) and WKY rats (D, E, F) before predator stimulation. As shown, one Wistar rat showed extremely abnormal behaviors in face-washing times, rearing times and total distance as indicated by red circles. We did not see any extremely abnormal WKY rats in this screening test.
